# William C. Barrett, M.D., D.D.S., M.D.S.

**Published:** 1903-10

**Authors:** 


### Obituary.

### WILLIAM C. BARRETT, M.D., D.D.S., M.D.S.

Dr. Barrett died at Nauheim, Germany, August 22, 1903, with heart complications. His death was not a surprise to his friends, although the hope was felt that he would live to return to this country. While his death occurred August 22, it was not known in Buffalo until September 2. This is not surprising to those familiar with the difficulties attending death in a foreign land and especially in Germany, when removal to the native land is deemed necessary. It is attended with innumerable and harrassing official supervision.

"Dr. Barrett was a son of Rev. William and Hannah Cheney (Tanner) Barrett, and was born on May 13, 1834, in Monroe County, N. Y. He was therefore in his seventieth year.

"After a thorough academic education at Kingsville Academy, Ohio, Carey Seminary, New York, and Yates Academy, New York, for some years he was a teacher in different literary institutions in the State of New York. In 1863 he began the study of medicine, but in 1864 he changed to that of dentistry, receiving the degree of Master of Dental Surgery in 1869. He began dental practice in the village of Warsaw, Wyoming County, N. Y., and remained there until the spring of 1876, when he moved to Buffalo, and in 1887 again took up the study of medicine in the Medical Department of the University of Buffalo, graduating with the degree of M.D. in 1880. He also attended lectures in the Pennsylvania College of Dental Surgery, in Philadelphia, and graduated with the degree of Doctor of Dental Surgery in 1881.

"After that time he was in the practice of his profession in the city of Buffalo. In 1885 he received the appointment of lecturer of Oral Pathology in the Medical Department of the University of Buffalo, his alma mater, and in 1890 was elected to the full professorship. In 1889 he was elected professor of Anatomy and Pathology in the Chicago College of Dental Surgery, and accepted after due consideration, but still continuing to maintain his residence in Buffalo. After that appointment he visited Chicago regularly for the purpose of delivering his lectures and giving the instruction belonging to his chair.

"Upon the organization of the Dental Department of the University of Buffalo, in 1891, Dr. Barrett was appointed professor of the Principles and Practice of Dentistry and Dental Pathology, and was elected dean of the faculty, which position he held at the time of his death. He was also one of the staff of the Buffalo General Hospital, holding the position of oral surgeon in that institution.

"From 1882 to 1888 he was editor of the *Independent Practitioner,* devoted to dental medicine and surgery. In 1888 it was sold and the editor retired from journalism, but in 1891 he again entered the field as editor of the *Dental Practitioner,* of Buffalo.

"Dr. Barrett was a member of the Medical Society of the County of Erie, of the Buffalo Medical and Surgical Association, and of the American Medical Association. He was a member of the International Medical Congress which met in London in 1881; an honorary vice-president of the International Medical Congress, Washington, 1887; and of the congress of 1890, which met in Berlin. He was a member of the International Dental Federation, that met at Stockholm, Sweden, August, 1902. He was president of the Dental Society of the State of New York in 1875 and 1876, and of the American Dental Association in 1886. He was a member of the American Microscopical Society and honorary member of many State and foreign professional associations. Dr. Barrett was a member of the Masonic Fraternity, belonging to Queen City Lodge, F. and A. M., and was a Knight Templar. He was also a musician, and was at one time in charge of Asbury church choir."

Dr. Barrett's tremendous energy naturally led him into many fields of human endeavor, as the foregoing abundantly illustrates. His ability as an editor was markedly illustrated in the two journals under his management. The editorial pages bristled with his ideas of what dentistry should do or leave undone, and, in the main, his effort was to advance his profession to a higher standard.

He was not a prolific writer upon medical or dental topics, yet some of his papers have a special value. He took a deep interest in comparative anatomy, and did very much to create an interest in this important branch.

In 1898 he gave to dentistry his "Oral Pathology and Practice." This was intended as a text-book for dental colleges, and, while very defective in the writer's estimation, met with a demand which required a second edition shortly thereafter. Dr. Barrett at the time of his death was engaged upon a History of Dentistry in New York. It is not known whether he had been able to complete this.

The last years of Dr. Barrett's life were spent in strenuous activities sufficient to break down any ordinary health. The chairmanship of the Foreign Relations Committee of the National Association of Dental Faculties required the possession of mental power not generally possessed. The position meant constant antagonisms both from within and from without,—within the circle of his colleagues and without in the diploma traffic which he unearthed in Illinois. Combined with this was the great labor in organizing the foreign work connected with this department. To fill such a position and avoid criticism was an impossibility, but this criticism was always resented by the chairman as an infringement upon the privileges of his committee. While this was a weakness, it may readily be forgotten and forgiven in view of the really great work Dr. Barrett did in this direction. He had a power of organization that few possess, and the results of his labor in this special direction will long remain an honor to him, and this, indirectly, will be reflected upon the association he so faithfully served.

Any review of such an active mentality must necessarily be imperfect. His restless spirit was ever alert for new fields to conquer, yet he never lost sight of the fact that his profession demanded that he give undivided devotion to its interests, and his compliance with this demand left but little time for things that smaller men delight in. To this untiring devotion may be ascribed the final breakdown of this strong and virile physical organism.

Dr. Barrett was the life and soul of the Dental Department of Buffalo University. His reputation, national and international, had much to do with bringing this school into prominence, and we are very apt to think, where this is the case, that the loss is a very serious setback, but it is well to remember that colleges are long-lived, and while they suffer in the loss of their great teachers, others will arise and the school will be the stronger. This, no doubt, will be the case with this department.

Dr. Barrett was married in 1857 to Amelia Harris Ryerse, of Port Ryerse, Ontario, who survives him. They had no children. Mrs. Barrett was with her husband in Germany at the time of his death.

